# Two strategies for the synthesis of the biologically important ATP analogue ApppI, at a multi-milligram scale

**DOI:** 10.3762/bjoc.11.237

**Published:** 2015-11-13

**Authors:** Janne Weisell, Jouko Vepsäläinen, Petri A Turhanen

**Affiliations:** 1 University of Eastern Finland, School of Pharmacy, Biocenter Kuopio, P.O. Box 1627, FIN-70211, Kuopio, Finland

**Keywords:** ApppI, ATP analogue, HPLC, purification, synthesis

## Abstract

Two strategies for the synthesis of the ATP (adenosine triphosphate) analogue ApppI [1-adenosin-5’-yl 3-(3-methylbut-3-enyl)triphosphoric acid diester] (**1**) are described. ApppI is an active metabolite of the mevalonate pathway and thus is of major biological significance. Chemically synthezised ApppI was purified by using triethylammonium bicarbonate as the counter ion in ion-pair chromatography and characterized by ^1^H, ^13^C, ^31^P NMR and MS spectroscopical methods.

## Findings

**Figure 1:**
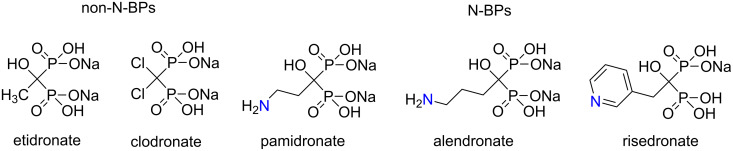
Examples of non-N-BPs and N-BPs.

Bisphosphonates (BPs), which are stable analogues of pyrophosphate occurring in cells, have been used for decades for the treatment of many kinds of bone related diseases, with osteoporosis probably being the most well-known indication [1–2]. BPs can be separated into two groups: non-nitrogen containing BPs (non-N-BPs) such as etidronate and clodronate, and nitrogen containing BPs (N-BPs) like pamidronate, alendronate and risedronate (Figure 1) [2]. BPs from these two groups have different mechanisms of action in the body. N-BPs, such as alendronate, inhibit the mevalonate pathway (MVP) in cells and promote the formation of ApppI, which has been demonstrated to induce apoptosis in osteoclasts [3]. Furthermore, ApppI has the ability to activate T cells together with isopentenyl diphosphate (IPP) [4]. ApppI has interesting properties and their clarification might explain many of the biological functions of N-BPs. Unfortunately, these studies are hampered by the very limited availability of ApppI.

There are only few methods described in the literature which can be used in the chemical synthesis of ApppI. One is the method Mönkkönen et al. [3] followed in their synthesis which has been first described by Eggerer and Lynen [5] and another method is the chemical synthesis of ApppI described by Vantourout et al. [6]. According to our earlier experiments following the procedure published by Eggerer and Lynen [5], the yield of the product after isolation was only 1–2%. Vantourout et al. [6] claimed that they could produce ApppI with a yield of 45% (mass of the product was not reported); they used a procedure adapted from Ryu and Scott [7] but their report did not contain any NMR characterization data, only HPLC and MS data were presented, and these are rather unsatisfactory methods with which to prove the purity of a product. They also stated that there was 5–10% ADP (adenosine diphosphate) as an impurity in some preparations and that it is possible that there may have been other impurities which could not be detected with HPLC and MS techniques.

**Scheme 1:**
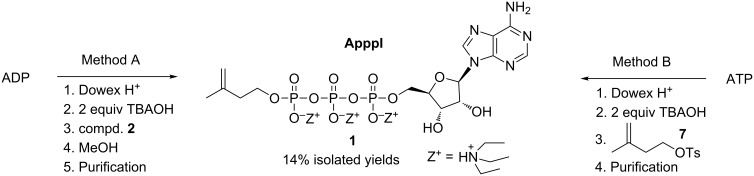
Synthesis of ApppI (**1**).

The starting point for the research presented in this paper was the urgent need to produce a supply of ApppI for different kinds of biological assays and as a reference compound for mass spectrometric analyses. We have received several requests to synthesize ApppI. Here we report two reasonable methods which can produce ApppI at a multi-milligram scale; one of these syntheses is a novel approach (method A, Scheme 1); the second is based on the method reported by Ryu and Scott [7] which was subsequently adapted by Vantourout et al. [6]; however, we provide full details of the approach (method B, Scheme 1).

**Scheme 2:**
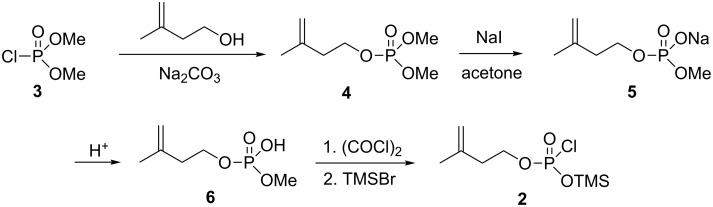
Synthesis of 3-methylbut-3-en-1-yl (trimethylsilyl) phosphorochloridate (**2**).

In method A, the bis(tetrabutylammonium) salt of ADP was first treated with in situ prepared 3-methylbut-3-en-1-yl (trimethylsilyl) phosphorochloridate (**2**) (see synthesis route in Scheme 2) followed by deprotection of the silyl group with MeOH to produce ApppI (**1**). It needs to be mentioned that the conversion of salt **5** into its acid form **6** was performed using HCl (aq), because the more common Dowex H^+^ resin treatment caused an unexpected side-reaction which led us to find a novel powerful tool to perform the organic addition and substitution reactions [8]. In method B, the bis(tetrabutylammonium) salt of ATP was treated with isopentenyl tosylate to produce ApppI. The crude products were purified by an ion-pair chromatographical approach, based on the method first reported by Ryu and Scott [7], using triethylammonium bicarbonate (TEAB) as the ion-pair reagent. In both methods, the reaction was performed at near to physiological temperature (35–40 °C). Interestingly, the yield of product **1** after purification was approx. 14%, irrespective of which method was used (method A or B); these yield amounts are very reasonable for this kind of compound which has two chemically and enzymatically labile P–O–P bonds. In fact, when the temperature was raised slightly, to only approx. 45–50 °C, the yields were lower probably due to the instability of ApppI at the elevated temperature. However, the purified ApppI (**1**) seems to be stable, since a NMR sample was stored in D_2_O in an NMR tube at room temperature and after approx. 1.5 years’ storage, the ^1^H and ^31^P NMR spectra were measured and no evidence of degradation was observed according to the spectra, i.e., it can be concluded that purified ApppI tris(triethylammonium) salt is highly stable against chemical hydrolysis at room temperature.

Since Vantourout et al. [6] did not report the amount of tetrabutylammonium used in their synthesis of ApppI, we tested whether mono tetrabutylammonium salts of ADP and ATP could be used as starting materials to produce ApppI (**1**) following both methods A and B, respectively, but either very little or no production of ApppI (**1**) at all could be detected according to the ^1^H and ^31^P NMR spectra measured from the crude products. In addition, other strategies for the preparation of ApppI (**1**) were tested but these methods led to the formation of very complicated mixtures of products.

It remain unclear for us why reasonably reported method(s) to prepare ApppI lack in the literature because methods to prepare ATP analogues have been reported [9–11]. We propose that one of the main reasons for this maybe the sensitivity of the isopentenyl group to addition reactions under mild reaction conditions [8] combined with the sensitivity of the two P–O–P bridges for hydrolysis. These points may explain at least partly the quite low 14% isolated yields for ApppI we reported in this paper.

## Conclusion

In conclusion, two feasible methods to synthesize the very important metabolite ApppI (**1**) have been described and for the first time, the methods are described in detail. ApppI is a rather interesting compound which is known to exert several unique biological effects and it is possible that novel properties await discovery. Now we can produce ApppI at a multi-milligram scale which will be important in advancing research into this interesting metabolite.

## Experimental

^1^H, ^31^P and ^13^C NMR spectra were recorded on a 500 MHz spectrometer operating at 500.1, 202.5 and 125.8 MHz, respectively. Dimethyl phosphorochloridate (**3**) is commercially available. 3-Methyl-3-buten-1-yl dimethyl phosphate (**4**) can be prepared as reported by Chen and Dale Poulter elsewhere [12]. Commercially available ADP mono sodium salt and ATP disodium salt were converted to the corresponding bis(tetrabutylammonium) salts by rapid treatment with Dowex H^+^ resin and the addition of 2 equiv of 40% tetrabutylammonium hydroxide in H_2_O. The ApppI tris(triethylammonium) salt was converted into the disodium salt by the same method except that 0.1 M NaOH solution was used (see additional ^13^C NMR spectrum in Supporting Information File 1). The solvent residual peak was used as a standard for ^1^H and ^13^C measurements in CDCl_3_ and CD_3_OD (7.26 ppm or 77.16 ppm for CDCl_3_ and 3.31 ppm or 49.00 ppm for CD_3_OD, respectively) [13], in D_2_O 4.79 ppm in ^1^H measurements and trimethylsilylpropionic acid sodium salt (TSP) in ^13^C measurements (0.00 ppm) and 85% H_3_PO_4_ was used as an external standard in the ^31^P measurements. The ^n^*J*_HP_ couplings were calculated from proton spectra and all *J* values are given in Hz. The ^n^*J*_CP_ couplings were calculated from carbon spectra with the coupling constants given in parenthesis as Hertz. Ion-pair chromatography was performed with a VP10 HPLC system using a Phenomenex Jupiter C18-column (250 × 10 mm) and with an MeCN gradient [0.1 M TEAB (triethylammonium bicarbonate) in H_2_O/0–37% MeCN gradient for 35 min] with a flow rate of 5 mL/min. The gradient started after 2.5 min loading of sample into column using 0.1 M TEAB in H_2_O. Mass spectra were recorded on a quadrupole time-of-flight mass spectrometer using electrospray ionization (ESI) in the positive ionization mode. The purity of the products was determined from ^1^H and ^31^P NMR spectra and was ≥95% unless stated otherwise.

**Procedure for the preparation of isopentenyl tosylate (7):** 3-Methyl-3-buten-1-ol (770 µL, 655 mg, 7.6 mmol) was dissolved in dry acetonitrile (10 mL), distilled pyridine (610 µL, 602 mg, 7.6 mmol) and tosyl chloride (1460 mg, 7.7 mmol) were added and the reaction mixture was stirred 4 hours at room temperature. The reaction mixture was evaporated to dryness and dissolved in diethyl ether (20 mL), filtered and then the ether was removed in vacuo*.* The crude product was purified by silica column chromatography using EtOAc/hexane (5:95) as eluent to give **7** (1.22 g, 67%) as a colorless oil. All ^1^H NMR data were comparable to those reported elsewhere [14]. ^1^H NMR (CDCl_3_) 7.79 (d, *J =* 8.0, 2H), 7.34 (d, *J =* 8.0, 2H), 4.79 (s, 1H), 4.67 (s, 1H), 4.13 (t, *J =* 7.0, 2H), 2.45 (s, 3H), 2.35 (t, *J =* 6.8, 2H), 1.66 (s, 3H).

**Procedure for the preparation of 3-methyl-3-buten-1-yl dimethyl phosphate (4):** Dimethyl phosphorochloridate **3** (2.68 g, 2.0 mL, 18.5 mmol) was dissolved in dry acetonitrile (15 mL), dry Na_2_CO_3_ (2.15 g, 20.3 mmol) and 3-methyl-3-buten-1-ol (2.25 mL, 1.91 g, 22.2 mmol) were added and the reaction mixture was stirred for 7 days at 50 °C. The reaction mixture was evaporated to dryness and dissolved in diethyl ether (20 mL), filtered and volatile compounds were removed in vacuo*.* The product **4** (822 mg, 23%) was present as a colorless oil. All NMR data were comparable to those reported elsewhere (except –OC*H*_3_ signals which was wrongly interpreted to be two singlets although those protons are coupled to phosphorus) [12]. ^1^H NMR (CDCl_3_) 4.84 (s, 1H), 4.77 (s, 1H), 4.16 (vq, 2H), 3.76 (d, ^3^*J*_HP_ = 11.5, 6H), 2.41 (t, *J =* 6.5, 2H), 1.77 (s, 3H); ^31^P NMR (CDCl_3_) δ 1.97.

**Procedure for the preparation of methyl (3-methylbut-3-en-1-yl) hydrogen phosphate (6):** Compound **4** (817 mg, 4.2 mmol) was dissolved in dry acetone (15 mL), dried NaI (567 mg, 3.8 mmol) was added and the reaction mixture was stirred for 24 h at 60 °C before it was evaporated to dryness. The residue washed twice with diethyl ether and dried in vacuo yielding compound **5** as a yellow solid (734 mg, 96%). Compound **5** (375 mg, 1.86 mmol) was suspended in acetone (6 mL) and 6 M HCl (310 µL, 1 equiv) was added and reaction mixture stirred for 5 min. The reaction mixture was filtered and the filtrate evaporated to dryness. The crude product was purified by silica column chromatography using EtOAc/MeOH (3:7) as the eluent producing **6** (249 mg, 75%) as a viscous oil. ^1^H NMR (CD_3_OD) 4.78 (s, 1H), 4.76 (s, 1H), 3.95 (vq, 2H), 3.57 (d, ^3^*J*_HP_ = 11.0, 3H), 2.35 (t, *J =* 7.0, 2H), 1.77 (s, 3H); ^31^P NMR (CD_3_OD) δ 4.67; ^13^C NMR (CD_3_OD) δ 143.7, 112.3, 65.1 (d, ^2^*J*_CP_ = 6.3), 53.0 (d, ^2^*J*_CP_ = 6.3), 39.8 (d, ^3^*J*_CP_ = 7.5).

**Procedure for the preparation of crude 1-adenosin-5'-yl 3-(3-methylbut-3-enyl) triphosphoric diester (ApppI) (1), Method A**: Oxalyl chloride (180 mg, 120 µL, 1.42 mmol) was dissolved in dry DCM (2 mL), one drop of DMF was added and the reaction mixture stirred at 45 °C. Compound **6** (248 mg, 1.38 mmol) was dissolved in dry dioxane (3 mL) and added in portions to the reaction mixture. After the additions were completed, the reaction mixture was stirred for 1 h at 45 °C before the reaction mixture was cooled to room temperature and trimethylsilyl bromide (325 mg, 280 µL, 2.12 mmol) was added and the reaction mixture stirred overnight at room temperature. The reaction mixture was evaporated to dryness and dissolved in dry acetonitrile (3 mL) and added in portions to a solution of acetonitrile (4 mL) in which ADP bis(tetrabutylammonium) salt (1.25 g, 1.38 mmol) was dissolved. After the addition was completed, the reaction mixture was stirred for 24 h at 40 °C before it was evaporated to dryness and the residue dissolved in MeOH (8 mL) and stirred overnight at room temperature. The reaction mixture was evaporated to dryness and crude product **1** (1.42 g) was obtained as hygroscopic solid. The HPLC purification run was performed (29 mg loaded amount) and compound **1** (3.8 mg, 13.5%) was obtained as a white hygroscopic solid after evaporation of appropriate fractions in vacuo. **Method B**: ATP bis(tetrabutylammonium) salt (187 mg, 0.189 mmol) was dissolved in dry acetonitrile (1 mL) and isopentenyl tosylate **7** (48 mg, 0.200 mmol) was added and the reaction mixture stirred for 24 h at 40 °C before the reaction mixture was evaporated to dryness and the residue washed with diethyl ether (3 mL) and dried in vacuo*.* The crude product **1** (204 mg) was obtained as a hygroscopic solid. Two HPLC purification runs were performed (20 mg and 24 mg loaded amounts) and compound **1** (tot. 4.8 mg, 14.2%) was obtained as a white hygroscopic solid after evaporation of the appropriate fractions in vacuo.

**1-Adenosin-5'-yl 3-(3-methylbut-3-enyl) triphosphoric diester (ApppI) tris(triethylammonium) salt (1):** White solid (very hygroscopic). ^1^H NMR (D_2_O) δ 8.59 (s, 1H), 8.32 (s, 1H), 6.18 (d, *J* = 6.0, 1H), 4.62–4.59 (m, 1H), 4.45–4.41 (m, 1H), 4.34–4.23 (m, 2H), 4.08–4.00 (m, 2H), 3.24 (q, *J* = 7.0, 16H, from triethylammonium salt), 2.30 (t, *J* = 6.5, 2H), 1.69 (s, 3H), 1.31 (t, *J* = 7.5, 24H, from triethylammonium salt). C=C*H_2_* signals and one “sugar” proton signal under HDO-line at 4.8. ^13^C NMR (D_2_O) δ 158.5, 155.7, 152.1, 146.2, 142.8, 121.6, 114.2, 89.6, 86.9 (d, ^2^*J*_CP_ = 9.3), 77.2, 73.3, 68.1 (d, ^3^*J*_CP_ = 5.0), 67.6 (d, ^2^*J*_CP_ = 6.1), 49.6, 40.6 (d, ^3^*J_CP_* = 7.3); ^31^P NMR (D_2_O) δ −10.36 d (^2^*J*_PP_ = 18.2), −10.74 d (^2^*J*_PP_ = 18.2), −22.54 t (^2^*J*_PP_ = 18.2); MS (ESI^+^): [M − H]^−^ calcd. for C_15_H_23_N_5_O_13_P_3_, 574.0505; found: 574.0517.

## Supporting Information

File 1 ^1^H, ^13^C and ^31^P NMR spectra for ApppI (**1**) and typical example of HPLC chromatogram of ApppI purification.
