# Supplementary material for: Two strategies for the synthesis of the biologically important ATP analogue ApppI, at a multi-milligram scale
Source: Beilstein J Org Chem. 2015 Nov 13;11:2189–93. doi: 10.3762/bjoc.11.237 (PMC4660979; doi:10.3762/bjoc.11.237)
Supplement: File 1 — 1H, 13C and 31P NMR spectra for ApppI (1) and typical example of HPLC chromatogram of ApppI purification. [file Beilstein_J_Org_Chem-11-2189-s001.pdf]

Supporting Information

for

Two strategies for the synthesis of the biologically important ATP analogue ApppI, at a multi-milligram scale

Janne Weisell, Jouko Vepsäläinen and Petri A. Turhanen*

Address: University of Eastern Finland, School of Pharmacy, Biocenter Kuopio, P.O. Box 1627, FIN-70211, Kuopio, Finland

Email: Petri A. Turhanen - petri.turhanen@uef.fi

*Corresponding author

^{1}H, ^{13}C and ^{31}P NMR spectra for ApppI (1) and typical example of HPLC chromatogram of ApppI purification

Table of Contents

^{1}H, ^{31}P and ^{13}C NMR spectra of ApppI (1) tris(triethylammonium) salt **S2**

^{13}C NMR spectrum of ApppI (1) disodium salt **S5**

Typical HPLC chromatogram of ApppI purification **S6**

Apppl

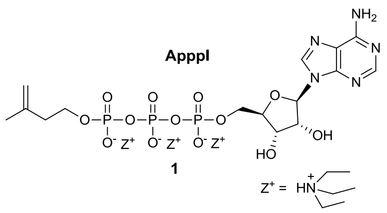

NH2
Z+ = HN

1

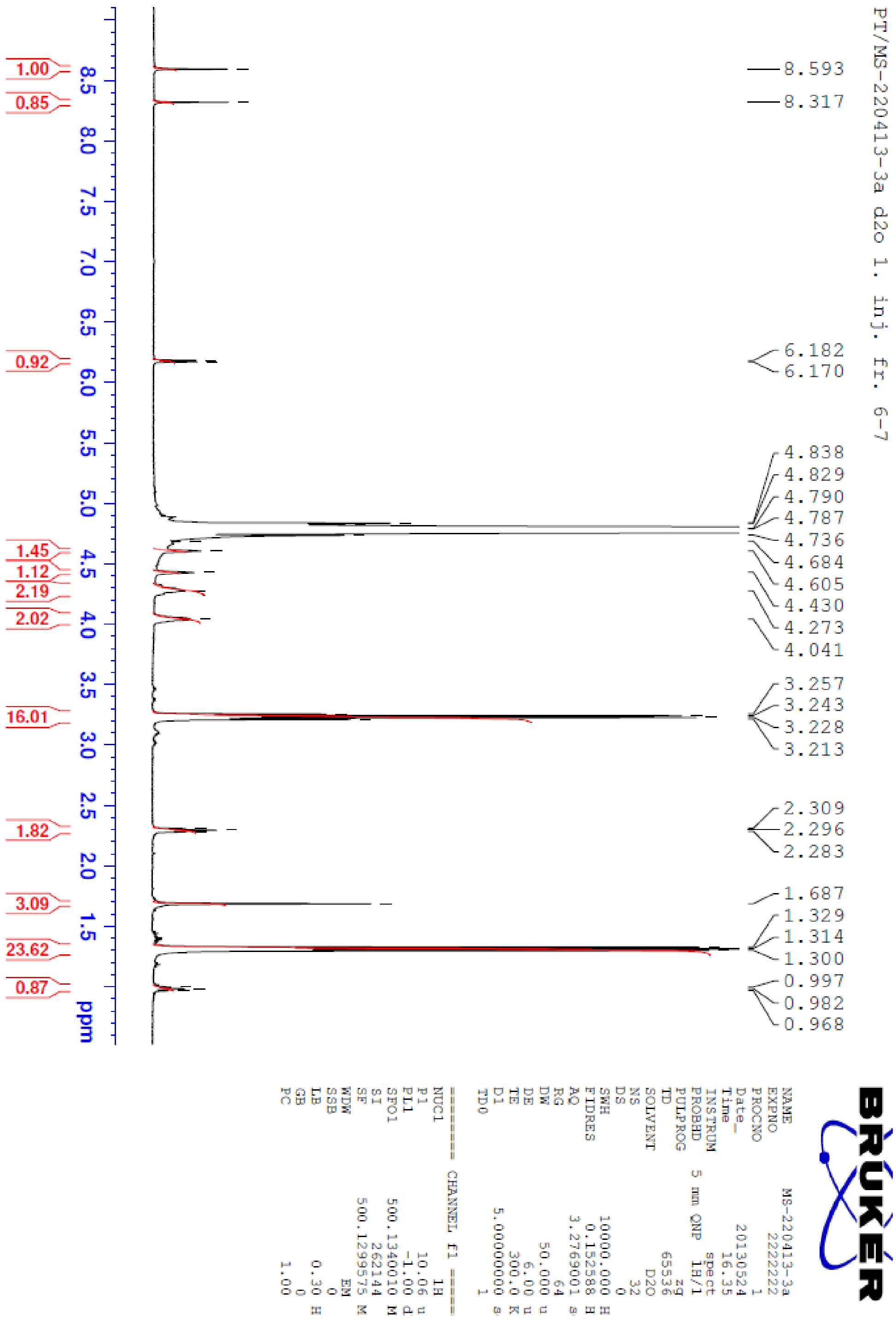

PT/MS-220413-3a d2o 1. inj. fr. 6-7
8.593
8.317
6.182
6.170
4.838
4.829
4.790
4.787
4.736
4.684
4.605
4.430
4.273
4.041
3.257
3.243
3.228
3.213
2.309
2.296
2.283
1.687
1.329
1.314
1.300
0.997
0.982
0.968
1.00
0.85
0.92
1.45
1.12
2.19
2.02
16.01
1.82
3.09
23.62
0.87
8.5 8.0 7.5 7.0 6.5 6.0 5.5 5.0 4.5 4.0 3.5 3.0 2.5 2.0 1.5 ppm
BRUKER
NAME MS-220413-3a
EXPNO 2222222
PROCNO 1
Date_ 20130524
Time 16.35
INSTRUM spect
PROBHD 5 mm QNP 1H/1
PULPROG zg
TD 65536
SOLVENT D2O
NS 32
DS 0
SWH 10000.000 Hz
FIDRES 0.152588 Hz
AQ 3.2769001 sec
RG 64
DW 50.000 usec
DE 6.00 usec
TE 300.0 K
D1 5.00000000 sec
TD0 1
======== CHANNEL f1 ========
NUC1 1H
P1 10.06 usec
PL1 -1.00 dB
SFO1 500.1340010 MHz
SI 262144
SF 500.1299575 MHz
WDW EM
SSB 0
LB 0.30 Hz
GB 0
PC 1.00

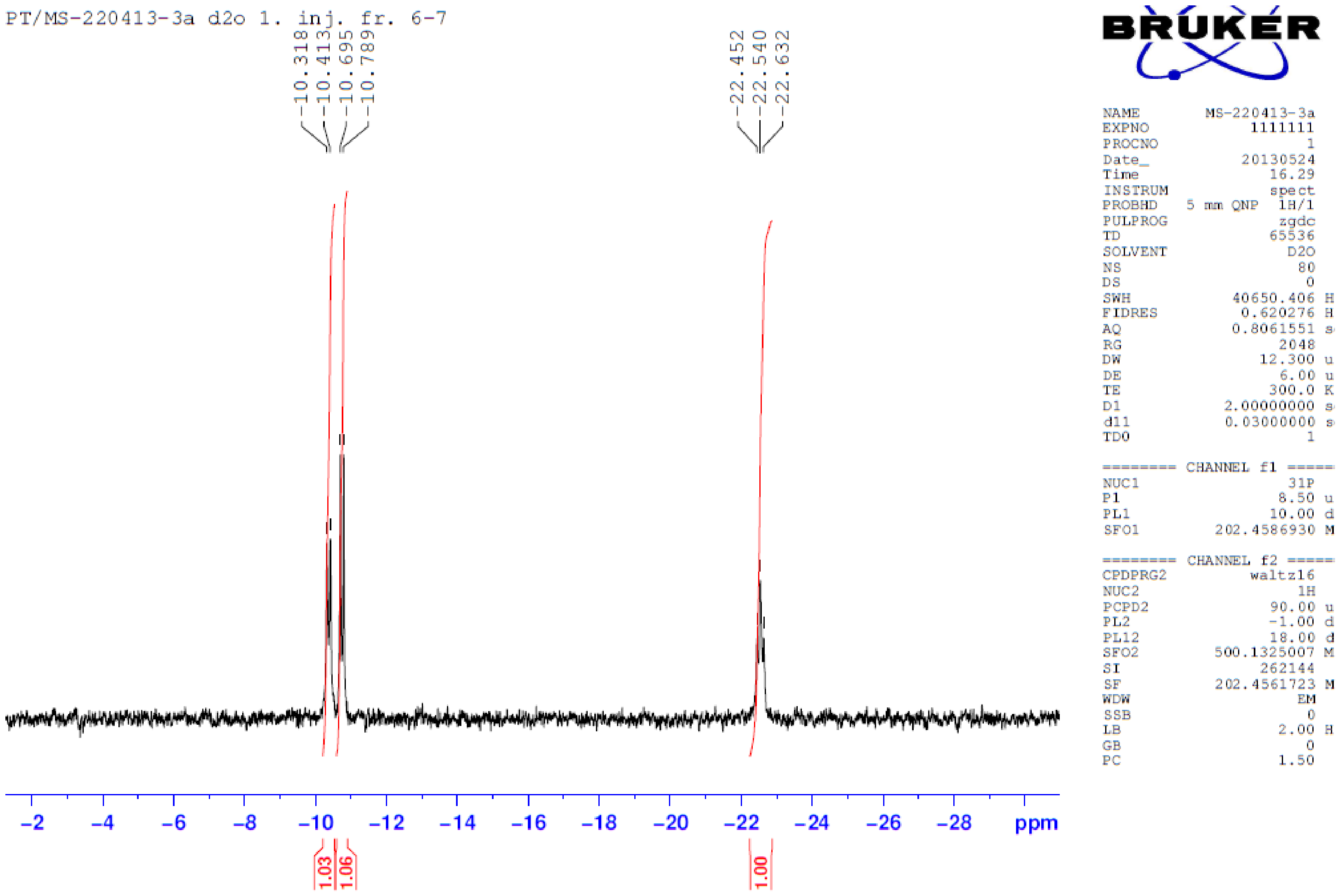
PT/MS-220413-3a d2o 1. inj. fr. 6-7
-10.318
-10.413
-10.695
-10.789
-22.452
-22.540
-22.632
-2
-4
-6
-8
-10
-12
-14
-16
-18
-20
-22
-24
-26
-28
ppm
1.03
1.06
1.00
NAME MS-220413-3a
EXPNO 1111111
PROCNO 1
Date_ 20130524
Time 16.29
INSTRUM spect
PROBHD 5 mm QNP 1H/1
PULPROG zgdc
TD 65536
SOLVENT D2O
NS 80
DS 0
SWH 40650.406 H
FIDRES 0.620276 H
AQ 0.8061551 s
RG 2048
DW 12.300 u
DE 6.00 u
TE 300.0 K
D1 2.00000000 s
d11 0.03000000 s
TD0 1
======== CHANNEL f1 =====
NUC1 31P
P1 8.50 u
PL1 10.00 d
SFO1 202.4586930 M
======== CHANNEL f2 =====
CPDPRG2 waltz16
NUC2 1H
PCPD2 90.00 u
PL2 -1.00 d
PL12 18.00 d
SFO2 500.1325007 M
SI 262144
SF 202.4561723 M
WDW EM
SSB 0
LB 2.00 H
GB 0
PC 1.50

PT/MS-220413-3a d2o tsp 1. inj. fr. 6-7

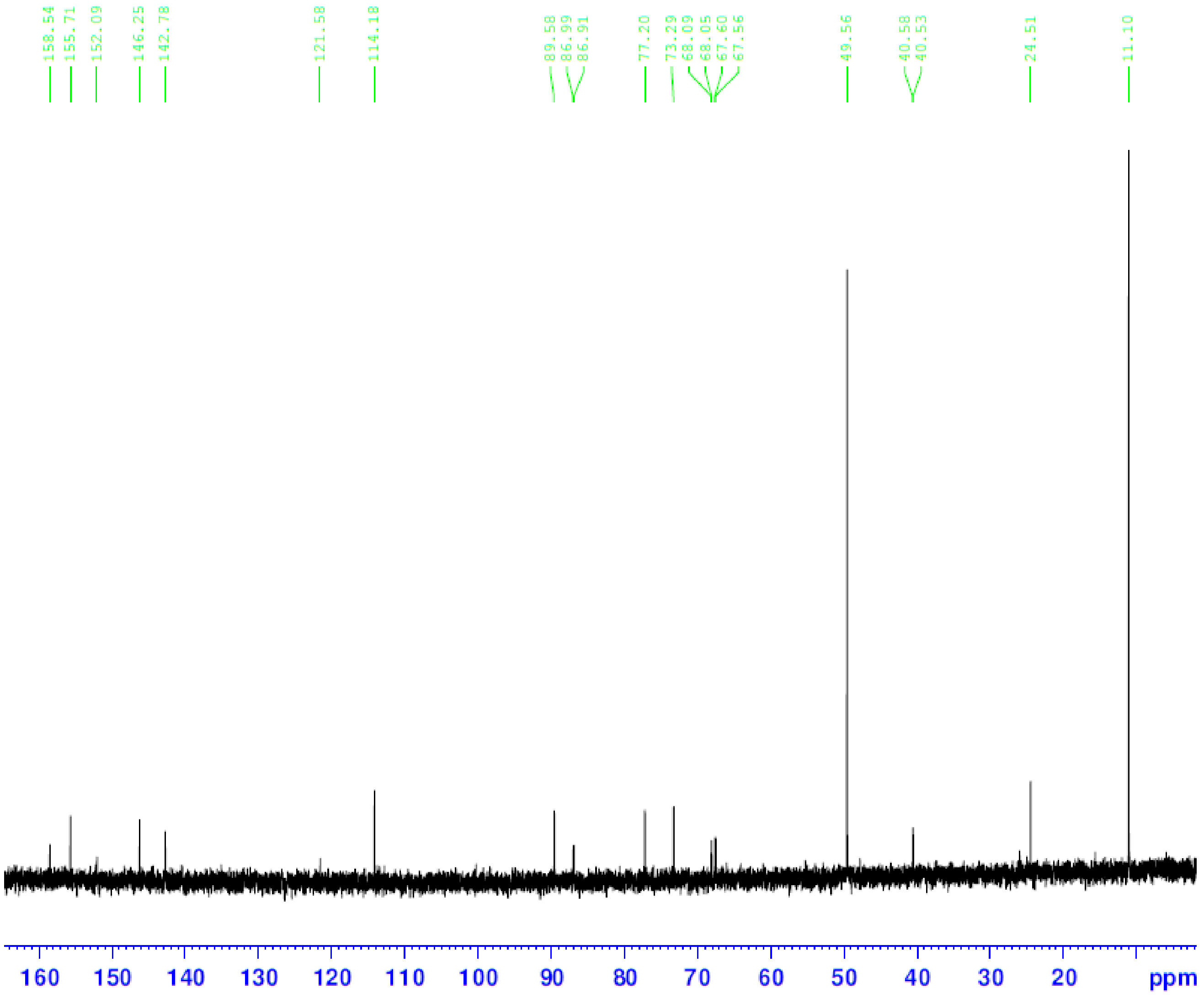

BRUKER

NAME	MS-220413-3a	
EXPNO	3333333	
PROCNO	1	
Date_	20130526	
Time	21.00	
INSTRUM	spect	
PROBHD	5 mm QNP 1H/1	
PULPROG	zgdc	
TD	131072	
SOLVENT	MeOD	
NS	8192	
DS	0	
SWH	30303.031	Hz
FIDRES	0.231194	Hz
AQ	2.1627545	sec
RG	8192	
DW	16.500	use
DE	6.00	use
TE	300.0	K
D1	20.00000000	sec
d11	0.03000000	sec
TD0	1	
======== CHANNEL f1 =======		
NUC1	13C	
P1	8.75	use
PL1	6.00	dB
SFO1	125.7715724	MHz
======== CHANNEL f2 =======		
CPDPRG2	waltz16	
NUC2	1H	
PCPD2	90.00	use
PL2	-1.00	dB
PL12	18.00	dB
SFO2	500.1322506	MHz
SI	524288	
SF	125.7572528	MHz
WDW	EM	
SSB	0	
LB	1.20	Hz
GB	0	
PC	0.90	

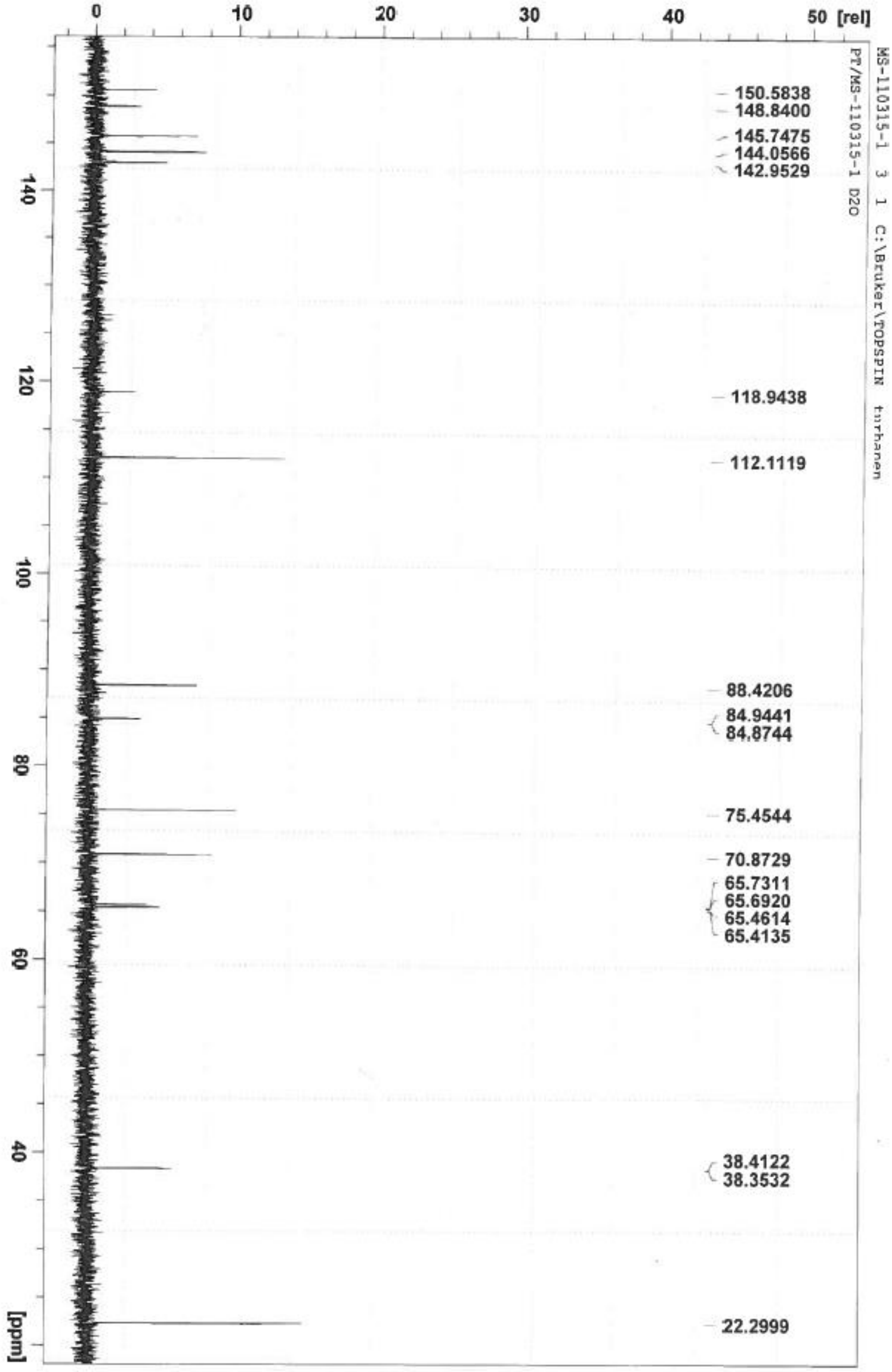
MS-110315-1 3 1 C:\Bruker\TOPSPIN turhanen
PT/MS-110315-1 D2O
150.5838
148.8400
145.7475
144.0566
142.9529
118.9438
112.1119
88.4206
84.9441
84.8744
75.4544
70.8729
65.7311
65.6920
65.4614
65.4135
38.4122
38.3532
22.2999
[ppm]
[rel]

```
Sample:   PTMS-220413-3 22mg
Run time: 3.12.2013 8:59:04

File:     C:\CLASS-VP\BMG\prep\data\ApppI\PTMS-220413-3 22mgU.dat
Method:   C:\CLASS-VP\BMG\prep\Methods\Jupiter C18 0.1MTEAB 37% gradientti ApppI puhd
46min.met
User:     Ale Närvänen
Analysed: 3.12.2013 9:42:26
```

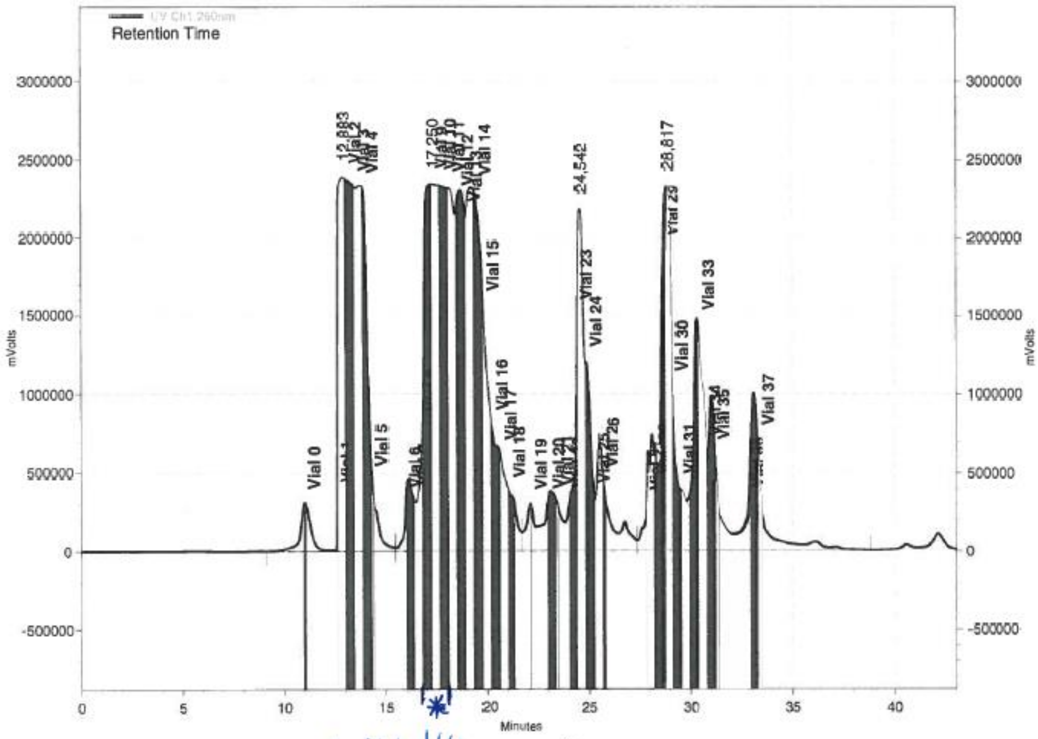

1-4 + 41-44 9.9059
8-10 5,1519 → ApppI
11-16 5,2023
